# Gaze behaviors during free viewing revealed differences in visual salience processing across four major psychiatric disorders: a mega-analysis study of 1012 individuals

**DOI:** 10.1038/s41380-024-02773-5

**Published:** 2024-10-11

**Authors:** Kenichiro Miura, Masatoshi Yoshida, Kentaro Morita, Michiko Fujimoto, Yuka Yasuda, Hidenaga Yamamori, Junichi Takahashi, Seiko Miyata, Kosuke Okazaki, Junya Matsumoto, Atsuto Toyomaki, Manabu Makinodan, Naoki Hashimoto, Toshiaki Onitsuka, Kiyoto Kasai, Norio Ozaki, Ryota Hashimoto

**Affiliations:** 1 Department of Pathology of Mental Diseases, National Institute of Mental Health, National Center of Neurology and Psychiatry, Kodaira, 184-8553 Japan; 2 Section of Brain Function Information, National Institute for Physiological Sciences, Okazaki, 444-8585 Japan; 3 Center for Human Nature, Artificial Intelligence, and Neuroscience (CHAIN), Hokkaido University, Sapporo, 060-0812 Japan; 4 Department of Rehabilitation, University of Tokyo Hospital, Tokyo, 113-8655 Japan; 5 Department of Psychiatry, Osaka University Graduate School of Medicine, Suita, 565-0871 Japan; 6 Medical Corporation Foster, Life Grow Brilliant Mental Clinic, Osaka, 531-0075 Japan; 7 Japan Community Health Care Organization, Osaka Hospital, Osaka, 553-0003 Japan; 8 Department of Neuropsychiatry, Graduate School of Medical Sciences, Kyushu University, Fukuoka, 812-8582 Japan; 9 Department of Psychiatry, Nagoya University Graduate School of Medicine, Nagoya, 466-8550 Japan; 10 Department of Psychiatry, Nara Medical University School of Medicine, Kashihara, 634-8521 Japan; 11 Department of Psychiatry, Hokkaido University Graduate School of Medicine, Sapporo, 060-8638 Japan; 12 NHO Sakakibara Hospital, Tsu, 514-1292 Japan; 13 Department of Neuropsychiatry, Graduate School of Medicine, The University of Tokyo, Tokyo, 113-8655 Japan; 14 The International Research Center for Neurointelligence (WPI-IRCN), The University of Tokyo Institutes for Advanced Study (UTIAS), Tokyo, 113-0033 Japan; 15 Pathophysiology of Mental Disorders, Nagoya University Graduate School of Medicine, Nagoya, 466-8550 Japan; 16 Institute for Glyco-core Research (iGCORE), Nagoya University, Nagoya, 464-8601 Japan

**Keywords:** Psychiatric disorders, Schizophrenia

## Abstract

Aberrant salience processing has been proposed as a pathophysiological mechanism underlying psychiatric symptoms in patients with schizophrenia. The gaze trajectories of individuals with schizophrenia have been reported to be abnormal when viewing an image, suggesting anomalous visual salience as one possible pathophysiological mechanism associated with psychiatric diseases. This study was designed to determine whether visual salience is affected in individuals with schizophrenia, and whether this abnormality is unique to patients with schizophrenia. We examined the gaze behaviors of 1012 participants recruited from seven institutes (550 healthy individuals and 238, 41, 50 and 133 individuals with schizophrenia, bipolar disorder, major depressive disorder and autism spectrum disorder, respectively) when they looked at stationary images as they liked, i.e., free-viewing condition. We used an established computational model of salience maps derived from low-level visual features to measure the degree to which the gaze trajectories of individuals were guided by visual salience. The analysis revealed that the saliency at the gaze of individuals with schizophrenia were higher than healthy individuals, suggesting that patients’ gazes were guided more by low-level image salience. Among the low-level image features, orientation salience was most affected. Furthermore, a general linear model analysis of the data for the four psychiatric disorders revealed a significant effect of disease. This abnormal salience processing depended on the disease and was strongest in patients with schizophrenia, followed by patients with bipolar disorder, major depressive disorder, and autism spectrum disorder, suggesting a link between abnormalities in salience processing and strength/frequency for psychosis of these disorders.

## Introduction

The aberrant salience hypothesis of psychosis proposes that an aberrant assignment of salience to the elements of one’s experience leads to delusion and hallucination [1]. This definition of salience involves not only motivational or incentive salience derived from emotion and motivation [2] but also perceptual salience derived from novelty and sensory features [3]. A brain imaging study in patients with schizophrenia revealed reduced signaling in the midbrain (the substantia nigra/ventral tegmental area) with additional regions depending on the form of salience such as novelty, negative emotion, targetness, and rarity/deviance [4]. Miyata [5] argued that different domains of salience are functionally connected with each other, although each domain has different neural underpinnings, and that they may be described by a common computational framework.

Various anomalies in gaze behaviors have been observed in association with psychiatric disorders [6, 7]. Patients with schizophrenia exhibit an abnormal scan path during simple free-viewing tasks in which a patient was asked to look at pictures or geometric drawings as he or she desired [8–12]. The gaze trajectory of schizophrenia patients is limited to a narrower area of the images than that of healthy individuals. The scan path is interpreted as the temporal trajectory of the image location to which the observer consciously or unconsciously pays attention [13, 14]. Since bottom-up image salience and top-down requirements for ongoing tasks are driving forces of attentional processing, abnormal scan paths during free viewing tasks might, at least in part, result from aberrant perceptual salience in patients with schizophrenia.

One method of examining the characteristics of perceptual salience is to analyze gaze behaviors using a computational model of bottom-up image-based salience [15, 16]. This model computes visual salience at each image location derived from low-level visual features. The gaze trajectories of humans and nonhuman primates during visual exploration behaviors have been analyzed using this model in previous studies [15–19]. Yoshida et al. [19] applied this approach in the analysis of gaze behaviors in schizophrenia patients and found that the saliency values at the gaze of schizophrenia were persistently higher during the viewing period compared with the healthy individuals, suggesting that visual salience is affected in schizophrenia patients, such that patients might be attracted more by bottom-up visual features than healthy individuals. They further suggested that visual salience defined by orientation features are affected in schizophrenia. Gaze behaviors are known to be affected in different psychiatric disorders [7, 20]. The main purpose of this paper is to determine whether the abnormality in visual salience suggested by a previous study is specific feature in schizophrenia or transdiagnostic feature observed also in other psychiatric disorders. Cross-disorder studies of intermediate phenotypes of psychiatric disorders provide insight into the differences in the pathophysiological mechanisms underlying the abnormalities associated with individual disorders [21–28].

In the present study, by extending our previous study [19], we aimed to understand the differences in affected visual salience derived from low-level visual features by Itti–Koch’s model across four major disorders, namely, unipolar and bipolar mood disorders, autism spectrum disorder and schizophrenia. Here, we compared visual exploration behaviors of individuals with different disorders during free-viewing tasks using a mega-analysis approach with a large sample set consisting of more than a thousand individuals recruited from multiple institutes that joined the Cognitive Genetics Collaborative Research Organization (COCORO) [29, 30].

## Materials and methods

### Subjects

A total of 1012 subjects from 7 COCORO sites participated in the study: 550 healthy controls, 238 individuals with schizophrenia (SZ), 41 individuals with bipolar disorder (BD), 50 individuals with major depressive disorder (MDD), and 133 individuals with autism spectrum disorder (ASD) (Table 1 and Supplementary Table S1). Subject inclusion and exclusion criteria are described in Supplementary method 1. Some of these participants had participated in prior eye movement studies [31].

**Table 1:** Demographic information of participants.

		HC		SZ		BD		MDD		ASD	
		Mean	SD	Mean	SD	Mean	SD	Mean	SD	Mean	SD
OSK	Age	35.0	16.4	35.3	12.9	–	–	53.3	13.2	27.4	7.2
	Sex (m/f)	126/114		29/38		–		2/6		10/4	
KYS	Age	34.0	9.3	36.3	12.5	43.4	9.3	45.6	11.8	34.1	8.2
	Sex (m/f)	43/34		38/31		10/6		10/12		8/0	
NCNP	Age	42.5	14.2	35.8	14.4	–	–	36.0	10.6	26.4	4.3
	Sex (m/f)	24/42		13/28		–		4/1		3/2	
UTH	Age	38.2	8.2	31.3	10.0	38.3	15.0	37.8	8.6	28.9	9.5
	Sex (m/f)	26/35		16/11		2/1		4/1		5/4	
NGY	Age	40.0	16.1	43.0	8.5	51.5	14.6	53.7	10.7	32.2	10.1
	Sex (m/f)	27/27		5/16		6/7		1/2		14/0	
NARA	Age	26.0	4.8	29.5	10.6	–	–	–	–	28.3	8.1
	Sex (m/f)	27/18		0/2		–		–		61/21	
HKK	Age	33.4	2.6	26.5	8.2	43.8	15.5	54.1	17.0	13.0	–
	Sex (m/f)	6/1		5/6		5/4		3/4		0/1	

*HC* healthy control, *SZ* schizophrenia, *BD* bipolar disease, *MDD* major depressive disorder, *ASD* autism spectrum disorder.

### Task and stimuli

The participants faced a 19-inch liquid crystal display monitor (1280 × 1024 pixels) placed 70 cm from the observer’s eyes. The visual stimuli were presented using the Psychophysics Toolbox extension [32] in MATLAB (MathWorks, Natick, MA, USA). Each trial began with the presentation of a fixation point at the center of the display. Once the participant fixated on the fixation spot for a random duration, the fixation spot disappeared and a test image appeared. The test image was presented for 8 s and then disappeared, signaling the end of trial. This trial was repeated for 20 times with 20 different images, the order of which was randomly determined for each participant. The participant was instructed to view the image as they liked, i.e., a free-viewing condition. The images were chosen from five categories: buildings, everyday items, foods, fractal patterns, and grayscale noise patterns (four images for each).

### Recording and preprocessing of eye movement data

The recording and preprocessing of eye movement data were performed as described previously [11]. The eye position and pupil area of the left eye were measured at 1 kHz using an EyeLink 1000 Plus (SR Research, Ontario, Canada). Eye position data (in degrees) were smoothed with a digital finite impulse response (FIR) filter (−3 dB at 30 Hz), and eye velocity and acceleration traces were derived from a two-point difference algorithm. Eye movement recordings were segmented into blink, saccade, and fixation periods. The detected saccades included both regular and microsaccades. Here, following previous papers on microsaccades during free viewing [33], saccades with amplitudes greater than one degree were defined as regular saccades.

### Computational models and saliency analysis

**Fig. 1:**
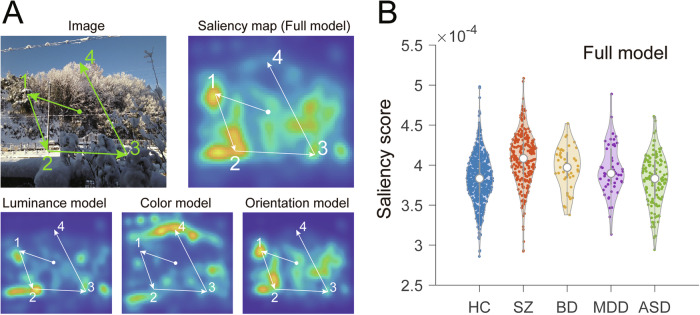
A schematic representation of saliency score computations and salience scores obtained from 1012 individuals. **A** The saliency map was generated using the Itti–Koch model. Visual salience was first defined for low-level visual feature models (color, luminance and orientation; the three panels at the bottom) and integrated into the full model of the saliency map (upper right panel). The gaze of a subject traveled over the image during a free-viewing trial, as schematically shown by the arrows (not real data). For the full model and each of the three low-level feature models, the salience values at gaze positions during the 8-sec presentation of an image were averaged and then further averaged over the 16 images. **B** Violin plot for the salience scores of the full model of the salience map calculated from 1012 individuals. The median and quantiles are shown as open circles and bars, respectively. HC healthy control, SZ schizophrenia, BD bipolar disorder, MDD major depressive disorder, ASD autism spectrum disorder.

The details of saliency map computations have already been described largely elsewhere (Figure S1 in Yoshida et al. [19]). To examine salience-guided eye movements, we used a validated computational model of visual attention and compared it with individual eye movements. The saliency maps for the test images were computed with the Itti–Koch’ standard saliency model for static images [15] implemented in the Graph-Based Visual Saliency (GBVS) toolbox for MATLAB [34]. The Itti–Koch model is a neurobiologically inspired model that computes visual salience at each location within the images derived from low-level image features. The net saliency map (referred to as the full model) was defined as the map that summed the saliency maps of the three low-level features with equal weights. Note that the saliency maps were created based purely on raw visual information. We evaluated the contribution of the full model and low-level visual features using the net saliency map and single-feature saliency maps, respectively. For each test image, we obtained a saliency map of 80 × 64 pixels. To treat the saliency maps as density maps, all the maps were normalized so that the sum of the saliency values of all the pixels was one. For each model of saliency map (full, color, luminance, and orientation models) of an image, the mean salience values at the endpoints of saccade made during presentation of the image were calculated. Then, the mean salience values for each model of saliency map were further averaged over the different images, referred to as the “salience score” for each model of saliency map. Thus, the salience score represented the mean saliency at the gazes of the participant. Sixteen of the 20 images were used for analysis, excluding those in the noise category since they had no color contrast. A schematic diagram of the quantification and salience scores for the full model of the 1012 individuals are shown in Fig. 1A, B, respectively. These analyses were carried out using MATLAB (MathWorks, NY). Code availability: the scripts used in the analysis will be available on reasonable request.

### Statistical analyses

The effect sizes of unbiased *d* [35] for differences in salience values between HCs and SZ patients were determined using a linear regression model within the institutes. The effect sizes for the full model and three low-level feature models were calculated, controlling for age and sex as covariates. A statistical analysis of the effect sizes across multiple institutes were performed applying the method used in meta-analyses (fixed effects model) for group comparisons. Power analysis was described in Supplementary Methods 2. Cross-disorder comparisons were also performed using general linear models for the full model and each of the three low-level feature models controlling for age, sex and institute. Post hoc pairwise comparisons of the marginal means were performed by applying Bonferroni correction. Additional correlation analyses were carried out between salience scores and PANSS scores for schizophrenia. Correlations analyses between salience scores and medication dosage were also performed (chlorpromazine equivalent for SZ, lithium carbonate for BD and imipramine equivalent for MDD). Bonferroni correction were performed for the correlation analyses. These analyses were carried out using Statistical Package for the Social Sciences (SPSS) 29.0 software (IBM Corp., Armonk, NY).

## Results

### Visual salience processing was affected in patients with schizophrenia

**Fig. 2:**
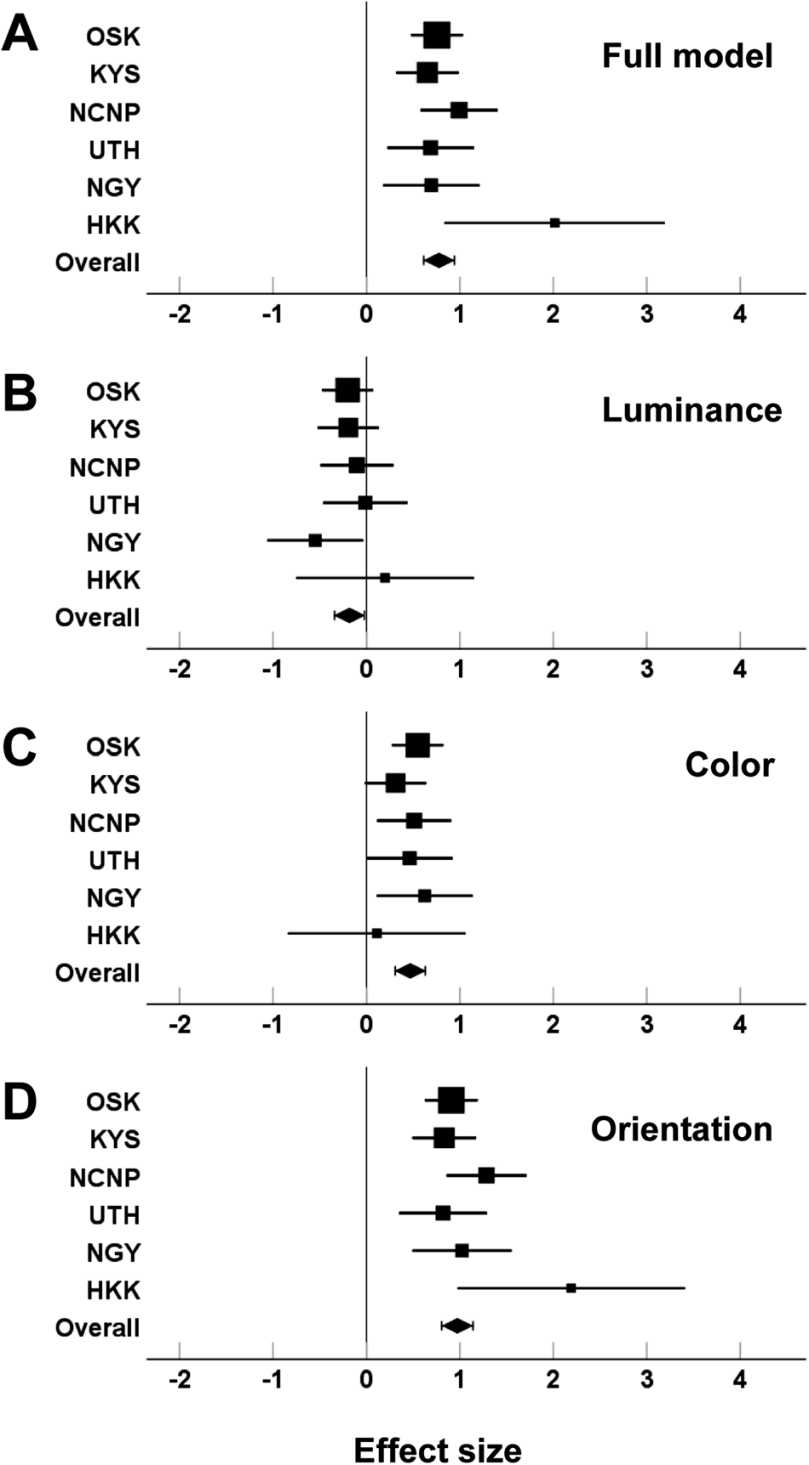
Forest plots of the effect-size of saliency scores obtained from patients with schizophrenia. **A** full model; **B** luminance feature model; **C** color feature model; **D** orientation feature model. The squares are the effect sizes at individual institutes, and the diamonds are the estimated overall effect sizes. Error bars indicate 95% confidence intervals.

According to the full model of the saliency map (Fig. 2A), the effect sizes obtained from individual institutes were all positive and ranged from 0.65–2.02. A statistical analysis with the fixed effects model performed across the six institutes showed that the estimated overall effect size (±SE) was 0.78 ± 0.08 (*p* = 1.96 × 10^−20^), indicating that individuals with schizophrenia looked at image locations with greater visual salience in the Itti–Koch model of saliency maps than did healthy individuals.

The statistical analyses with the fixed effects model performed on salience scores for the three low-level features also showed a significant effect in patients with schizophrenia. For the luminance feature (Fig. 2B), the effect sizes obtained from individual institutes were positive or negative depending on the institute and ranged from −0.55 to 0.20. The estimated overall effect size was −0.18 ± 0.08 (*p* = 0.03). For the color feature (Fig. 2C), the effect sizes obtained from individual institutes were positive and ranged from 0.11–0.62. The estimated overall effect size was 0.47 ± 0.08 (*p* = 1.08 × 10^−8^). The effect sizes for the orientation feature obtained from individual institutes were all positive and ranged from 0.82–2.20 (Fig. 2D). The estimated overall effect size was 0.97 ± 0.09 (*p* = 4.45 × 10^−30^). Thus, the orientation features had the highest overall effect size.

### Schizophrenia showed the largest affected visual salience among the four major psychiatric disorders

**Fig. 3:**
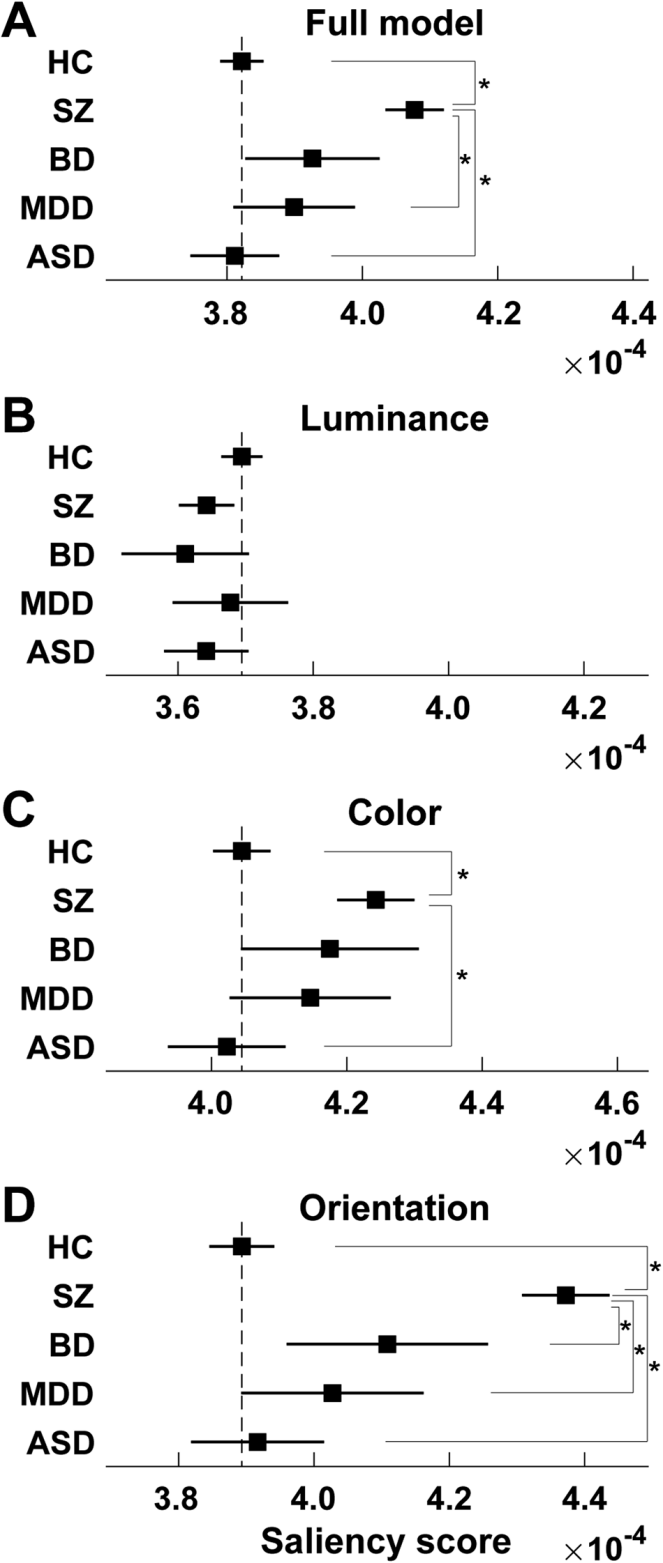
Estimated marginal means of the salience scores (aged 35.75 years) across four major psychiatric diseases obtained in the general linear model analyses. **A** full model; **B** luminance feature model; **C** color feature model; **D** orientation feature model. The broken lines indicate the marginal means of the healthy individuals. Asterisks indicate significant differences at *p* < 0.05 according to pairwise comparisons after Bonferroni adjustment for multiple comparisons. Error bars indicate 95% confidence intervals.

A general linear model analysis revealed a significant main effect of diagnostic group on visual saliency in the salience scores of the full model (*p* = 2.84 × 10^−21^). The estimated marginal mean of the salience score was the largest in patients with schizophrenia (Fig. 3A). Post hoc pairwise comparisons based on the estimated marginal mean showed significant differences between patients with schizophrenia and HCs (*p* = 1.94 × 10^−22^), patients with MDD (*p* = 3.78 × 10^−3^) and patients with ASD (*p* = 1.74 × 10^−9^). Further analyses of the three low-level features demonstrated significant group effects on the salience scores for the color (*p* = 1.24 × 10^−7^) and orientation features (*p* = 2.51 × 10^−32^) whereas no significant effect for luminance feature (*p* = 0.10). Post hoc pairwise comparisons based on the estimated marginal means for each low-level feature are shown in Fig. 3B–D. For the color feature, a significant difference was found between patients with schizophrenia and HCs (*p* = 2.85 × 10^−8^) and patients with ASD (*p* = 5.73 × 10^−4^) (Fig. 3C). For the orientation feature, the pairwise comparisons revealed significant differences between patients with schizophrenia and patients in all the other diagnostic groups (HC, *p* = 6.91 × 10^−34^; BD, *p* = 1.50 × 10^−2^; MDD, *p* = 4.43 × 10^−5^; ASD, *p* = 3.25 × 10^−12^; Fig. 3D). No significant association was detected after Bonferroni correction between saliency scores and symptom scales (Supplementary Table S2) and between saliency scores and medication dosages (Supplementary Table S3).

## Discussion

Analysis of visual salience during free viewing is a useful method for detecting psychiatric or neurological disorders [36, 37]. The current findings provide more evidence of the successful application of the computational salience model to the analysis of eye movements in participants with psychiatric disorders. In the present study, we found abnormalities in visual salience processing using the computational saliency map model, which is known to be biologically plausible in which the neuronal structures and functions that are known to exist in the visual system, such as Gabor filters and center-surround inhibitions are used to build the model. In our previous study, we found that patients with schizophrenia tended to focus on image positions with greater salience than did healthy individuals [19]. Among the low-level component features, individuals with schizophrenia seemed to be most attracted to orientation salience. These results were obtained using the data of approximately 300 individuals recruited at a single institute. Here, we confirmed these primary findings through the use of a much larger sample recruited from multiple institutes that joined a long-term, large-scale collaboration involving eye movement studies in the COCORO consortium in Japan. We further demonstrated that visual salience for the other low-level features might also be affected in schizophrenia patients, although it was much weaker than orientation salience was. Notably, cross-disorder comparisons showed that the schizophrenia group tended to focus on salient locations in the images compared with those of the other patient groups.

### Aberrant perceptual salience in schizophrenia patients

The present results demonstrated greater salience scores for the full model in schizophrenia patients than in healthy individuals. The data from the individual institutes consistently showed positive effect sizes, and the subsequent analysis across multiple institutes revealed that the overall effect was statistically significant. These results strongly support the previous findings of Yoshida et al. [19]. Larger salience scores indicated that the subjects tended to direct their gaze at image locations with higher bottom-up image feature salience according to Itti and Koch’s theory. Thus, our previous and present findings consistently suggest that perceptual salience in visual processing is affected in individuals with schizophrenia, consistent with the aberrant salience hypothesis of psychosis [1–5]. Similar findings were observed for the salience scores for the orientation feature, which showed the largest overall effect size. This result is also consistent with the findings of our previous study [19]. Interestingly, previous studies have demonstrated that orientation processing is affected in schizophrenia [38, 39] and that the early visual cortex is involved in changes in contextual modulation in schizophrenia [40–42], consistent with the present findings. Besides, the effect of orientation salience can explain impairment in contour integration in schizophrenia [39] because contour integration can be distracted by aberrant processing of orientation salience. Here, we found that the overall effect sizes of salience scores for the other low-level features were significantly different from zero, which was not detected in our previous study. Since the effect sizes for color and luminance salience were less than 0.5 and 0.2, respectively, these additional findings might be due to increased detectability resulting from increased sample sizes. However, these overall effect sizes were approximately half or one-fifth of the overall effect size of orientation salience. Thus, the present results suggest that, among the three low-level features, processing orientation salience is most severely affected in patients with schizophrenia. In our previous discussion [19], we have argued that processing for orientation salience and for luminance processing resemble those in the early visual cortex and lateral geniculate nucleus (LGN) and that processing in the early visual cortex might be affected in schizophrenia. The present findings are also in line with this argument.

### Cross-disorder comparisons

Mental health conditions lie on a spectrum with partially overlapping causes and symptoms [43]. Previous findings demonstrated that gaze behaviors during visual exploration tasks in individuals with various psychiatric disorders, including schizophrenia, bipolar disorder, major depressive disorder, and autism spectrum disorders, were different from those of healthy individuals [6–8, 20, 44–49]. The present cross-disorder study investigated the differences in bottom-up image salience during a free-viewing task among individuals with different psychiatric disorders. Overall, our results showed that the highest salience score was observed in patients with schizophrenia, followed by patients with bipolar disorder, major depressive disorder and autism spectrum disorder. This order is consistent with the empirical order of severity or frequency of psychosis (e.g., delusions, hallucinations, disordered thoughts) among the four psychiatric disorders [43]. Interestingly, the overall extent of alterations was the largest in patients with schizophrenia, followed by patients with bipolar disorder and major depressive disorder; patients with autism spectrum disorder tended to have fewer alterations in subcortical volume, cortical thickness and white matter microstructural abnormalities than patients with schizophrenia and bipolar disorder [26, 27, 50]. The order of abnormalities in visual salience was similar to the findings of neuroimaging studies. Furthermore, the genetic correlation, calculated using common single-nucleotide polymorphisms, was high between schizophrenia and bipolar disorder; moderate between schizophrenia and major depressive disorder and between bipolar disorder and major depressive disorder; and low between schizophrenia and autism spectrum disorder [51–53].

### Comparisons with previous studies

In a previous study, gazes of participants with ASD showed a difference in pixel-based salience defined by low-level visual features compared with healthy individuals [49]. On the other hand, the present results demonstrated no marked difference in salience scores for any of the features in individuals with ASD. The individuals with ASD who participated in the present study were not restricted by age, sex or intellectual performance, whereas the previous study was restricted to high-functioning individuals with ASD. Furthermore, the methods of analysis were different; here, we used a relatively simple model to compute salience scores, whereas previous studies adopted a much more elaborate model involving not only bottom-up image salience but also semantic, object-based salience features. These differences might explain the discrepancies between the findings.

In terms of the predictive coding/Bayesian brain hypothesis, both schizophrenia and ASD are explained by difficulties in evaluating prediction errors in sensory signals [54, 55]. Distinguishing the two psychiatric disorders from a computational perspective is an ongoing challenge. By extending the concept of visual salience to Bayesian surprise [56] and treating visual salience as spatial outliers from the current belief on what we see, saliency analysis could help resolve this issue. According to our present findings, schizophrenia patients showed the most affected processing of visual salience of bottom-up image features, whereas ASD patients showed marked contrast with schizophrenia patients, suggesting a different mechanism for evaluating prediction errors in sensory signals between the two psychiatric disorders.

### Discrimination among psychiatric disorders

Several lines of evidence have suggested that eye movement characteristics are effective at distinguishing schizophrenia patients from healthy controls [9–11, 57–61]. Gaze behaviors during free viewing were suggested to be most useful for discrimination [9, 11, 60]. Here, we showed that the salience score for the orientation feature in schizophrenia patients was significantly different from that in patients with other psychiatric disorders. The differences in salience scores might be useful in distinguishing between individuals with schizophrenia and other psychiatric disorders. However, it should be further investigated in the future. In the field of neurology, Tseng et al. [37] developed a technique for high-throughput classification of clinical populations, namely, attention-deficit/hyperactivity disorder, fetal alcohol spectrum disorder and Parkinson’s disease, using natural viewing eye movements.

### Limitations

This study has several limitations. First, the participants in the current study were mainly adults. Thus, there are no data showing whether the current findings are generalizable to children or elderly individuals. Second, the current study included many medicated patients, and it is not clear to what extent the effects are due to the disease, drug treatment, or both though no significant associations were found here (supplementary Table S3). To address this issue, studies of drug-free patients or, more ideally, drug-naïve patients are needed. In addition, to reveal the effects of psychotropic drug use on behavior, longitudinal, interventional studies comparing behaviors before and after use of specific medications are needed. Third, in addition to salience processing, its association with psychiatric symptoms should be examined in a cross-disorder fashion. In the present study, our data on symptoms are limited to diagnosis-specific rating scales; for example, we obtained Positive and Negative Syndrome Scale (PANSS) for schizophrenia patients, Hamilton Rating Scale for Depression (HAMD) for major depressive disorder patients. In the future, it will be necessary to conduct evaluations using cross-disorder rating scales.

In conclusion, we obtained strong and robust evidence for affected visual salience in patients with schizophrenia. The degree to which visual salience was affected depended on the psychiatric disorder; i.e., the strongest effects were observed for schizophrenia, followed by bipolar disorder, major depressive disorder, and autism spectrum disorder, suggesting a link between abnormalities in salience processing and strength/frequency for psychosis of these disorders. This finding is consistent with the aberrant salience hypothesis of psychosis.

## Supplementary information

Supplementary Information

## Data Availability

The data generated during and/or analyzed during the current study are not publicly available due to ethical reasons but are available from the corresponding author on reasonable request.
