# Supplementary material for: Gaze behaviors during free viewing revealed differences in visual salience processing across four major psychiatric disorders: a mega-analysis study of 1012 individuals
Source: Mol Psychiatry. 2024 Oct 11;30(4):1594–600. doi: 10.1038/s41380-024-02773-5 (PMC11919774; doi:10.1038/s41380-024-02773-5)
Supplement: Supplementary file 1 — Supplementary Information [file 41380_2024_2773_MOESM1_ESM.doc]

**Supplementary Information**

**Supplementary information for “Gaze behaviors during free viewing revealed differences in visual salience processing across four major psychiatric disorders: a mega-analysis study of 1012 individuals”**

**Supplementary method 1:** **Subject inclusion and exclusion criteria by site**

The data used in this study were collected at seven separate study sites: Osaka University (OSK), Kyushu University (KYS), the National Center for Neurology and Psychiatry (NCNP), University of Tokyo (UTH), Nagoya University (NGY), Hokkaido University (HKK) and Nara Medical University. In brief, all the subjects had no history of neurological or medical conditions that could influence the central nervous system, had no history of ophthalmological disease, and had normal or corrected-to-normal vision. The specific recruitment criteria for each site are described below.

All subjects at the Osaka site had normal or corrected-to-normal vision, were not biologically related and were of Japanese descent. Subjects were excluded if they had neurological or medical conditions that could influence the central nervous system, such as atypical headache, head trauma with loss of consciousness, chronic lung disease, kidney disease, chronic hepatic disease, thyroid disease, active cancer, cerebrovascular disease, epilepsy, seizures, substance-related disorders, mental retardation, or ophthalmological disease. Patients were recruited from Osaka University Hospital, and each patient was diagnosed by two or more trained psychiatrists according to the Diagnostic and Statistical Manual of Mental Disorders, Fourth Edition (DSM-IV) criteria based on the Structured Clinical Interview for DSM-IV (SCID)^1^. Healthy participants were recruited through local advertisements at Osaka University. These subjects were evaluated using the nonpatient version of the SCID^2^ to exclude individuals who had current or past contact with psychiatric services or who had taken psychiatric medications.

All subjects recruited at the Kyushu site were of Japanese origin, were physically healthy and had normal or corrected-to-normal vision at the time of the study. Subjects with neurological illness or major head trauma, electroconvulsive therapy, alcohol or drug dependence, alcohol or drug abuse within the past 5 years, or ophthalmological disease were excluded. All subjects with schizophrenia were recruited from Kyushu University Hospital and were diagnosed by at least two trained psychiatrists according to criteria from the DSM-IV using the SCID and medical records. Healthy controls were screened using the nonpatient version of the SCID; exclusion criteria included a history of Axis I psychiatric disorders in the subjects themselves or among their first-degree relatives.

All subjects recruited at the NCNP site had normal or corrected-to-normal vision, were not biologically related and were of Japanese descent. Subjects were excluded if they had neurological or medical conditions that could influence the central nervous system, such as atypical headache, head trauma with loss of consciousness, chronic lung disease, kidney disease, chronic hepatic disease, thyroid disease, active cancer, cerebrovascular disease, epilepsy, seizures, substance-related disorders, mental retardation, or ophthalmological disease. Patients were recruited from NCNP Hospital, and each patient was diagnosed by two or more trained psychiatrists according to the DSM-IV criteria based on the SCID. Healthy participants were recruited through local advertisements at NCNP. These subjects were evaluated using the nonpatient version of the SCID to exclude individuals who had current or past contact with psychiatric services or who had taken psychiatric medications.

All participants at the Tokyo site had normal or corrected-to-normal vision. Individuals with schizophrenia and healthy comparison subjects were recruited at the University of Tokyo Hospital. Schizophrenia was diagnosed according to DSM-IV criteria by a trained psychiatrist. Healthy controls were screened for psychiatric disorders using a semi-structured interview. Participants were excluded if they had current or past neurological illness, ophthalmological disease leading to decreased visual acuity, loss of consciousness for more than 5 minutes, previous substance abuse or dependence based on clinical histories.

Subjects recruited at the Nagoya site had normal or corrected-to-normal vision. Subjects were excluded if they had physical conditions that might influence the central nervous system or ophthalmological disease. Patients with schizophrenia were recruited from Nagoya University Hospital and affiliated psychiatric hospitals and diagnosed by trained psychiatrists according to the DSM-IV criteria. Healthy participants were recruited from the local community, hospital staff, and university students at Nagoya University and affiliated psychiatric hospitals. Healthy subjects were evaluated using the nonpatient version of the SCID, and individuals who had current or past contact with psychiatric services or who had taken psychiatric medications were excluded.

Participants at the Hokkaido site had normal or corrected-to-normal vision. Participants were excluded if they had physical conditions that could influence the central nervous system. Patients were recruited from the Hokkaido University Hospital. All patients were diagnosed by psychiatrists with at least 6 years of clinical experience according to the DSM-IV or DSM-5 criteria. Healthy participants were recruited through local advertisements at Hokkaido University. All healthy subjects were screened to exclude any psychiatric disorders using the Japanese version of the Mini-International Neuropsychiatric Interview (M.I.N.I.) ^3^ by trained psychiatrists.

At the Nara site, patients were recruited at the outpatient clinic of the Department of Psychiatry, Nara Medical University Hospital and the affiliated psychiatric clinic. They were diagnosed by two trained psychiatrists based on the DSM-V and the Japanese version of the Autism Diagnostic Observation Schedule-Second Edition (ADOS-2)^4^. The healthy subjects included students and hospital and university staff of Nara Medical University with no history of psychiatric, neurological, or developmental disorders as assessed based on the M.I.N.I. We also estimated the full-scale intelligence quotient (IQ) of each participant using the similarities and symbol search subsets of the Wechsler Adult Intelligence Scale-Third Edition^5, 6^. All subjects had normal or corrected-to-normal vision.

This study was performed in accordance with the World Medical Association’s Declaration of Helsinki and was approved by the Research Ethical Committee of each institution. After the full explanation of the study procedures, all participants provided written consent. Anonymity was preserved for all participants.

**Supplementary Methods 2: Power analysis**

Power analyses were conducted according to Mastumoto et al.^8^ at a power of 0.80 and a one-tailed significance level of 0.05 with G*Power version 3.1.9.7 to estimate the sample sizes required to detect the effect sizes. Number of subjects needed in each group for the given effect sizes in an analysis of saliency scores for the group comparisons between HC and SZ are 22 (d = 0.78, the full model), 57 (d = 0.47, the color model), 383 (d = -0.18, the luminance model) and 18 (right, d = 0.97, the orientation model).

Supplementary Table S1. Participant demographics

|  | HC | | |  | SZ | | |  | BD | | |  | MDD | | |  | ASD | | |
| --- | --- | --- | --- | --- | --- | --- | --- | --- | --- | --- | --- | --- | --- | --- | --- | --- | --- | --- | --- |
|  | N | mean | SD |  | N | mean | SD |  | N | mean | SD |  | N | mean | SD |  | N | mean | SD |
| Age | 550 | 35.8 | 14.3 |  | 238 | 35.4 | 12.6 |  | 41 | 45.7 | 13.2 |  | 50 | 46.8 | 13.3 |  | 133 | 28.8 | 8.4 |
| Sex (male/female) | 550 | 279/271 | |  | 238 | 106/132 | |  | 41 | 23/18 | |  | 50 | 24/26 | |  | 133 | 101/32 | |
| Education (years) | 543 | 15.3 | 2.3 |  | 236 | 13.6 | 2.4 |  | 41 | 14.6 | 2.5 |  | 49 | 13.8 | 2.5 |  | 131 | 14.1 | 2.4 |
| Onset age | - | - | - |  | 234 | 23.6 | 9.0 |  | 37 | 30.0 | 9.4 |  | 44 | 36.9 | 14.2 |  | - | - | - |
| Duration of illness (years) | - | - | - |  | 234 | 11.7 | 10.6 |  | 37 | 16.1 | 9.0 |  | 44 | 10.9 | 8.3 |  | - | - | - |
| Estimated IQ | 472 | 107.9 | 12.0 |  | 219 | 88.6 | 14.3 |  | 35 | 97.6 | 13.4 |  | 45 | 99.6 | 12.8 |  | 123 | 100.9 | 15.0 |
| Premorbid IQ | 532 | 108.8 | 7.5 |  | 225 | 102.0 | 9.8 |  | 35 | 107.2 | 8.6 |  | 46 | 106.0 | 9.9 |  | 123 | 107.7 | 9.0 |
| Work hours (hours/week) | 478 | 41.5 | 17.8 |  | 212 | 10.9 | 14.9 |  | 35 | 16.5 | 18.6 |  | 43 | 15.5 | 18.6 |  | 126 | 21.9 | 21.7 |

The estimated IQ was calculated using two WAIS-III subtests (Similarities and Symbol Search). This short form was developed to estimate full-scale IQ to reduce testing time while considering the predictability of full-scale IQ and its relation to functional outcomes ^5,6^. The premorbid IQ was estimated using the JART-25 (a short form of the JART) ^7^.

Supplementary Table S2. Correlation between saliency scores and PANSS scores in patients with schizophrenia

| Saliency map model |  | positive | negative | general | total |
| --- | --- | --- | --- | --- | --- |
| Full | *r* | 0.01 | -0.01 | -0.07 | -0.04 |
|  | (*p*) | (1.00) | (1.00) | (1.00) | (1.00) |
| Luminance | *r* | -0.10 | -0.17 | -0.16 | -0.16 |
|  | (*p*) | (1.00) | 0.22 | 0.32 | 0.32 |
| Color | *r* | 0.00 | -0.05 | -0.05 | -0.04 |
|  | (*p*) | (1.00) | (1.00) | (1.00) | (1.00) |
| Orientation | *r* | 0.06 | 0.08 | 0.00 | 0.04 |
|  | (*p*) | (1.00) | (1.00) | (1.00) | (1.00) |

*Only Bonferroni-corrected p-values are presented. Raw p-values were multiplied by 16 for correlations between saliency scores and symptom scales. N=209.

Supplementary Table S3. Correlation between saliency scores and medication dose in patients with schizophrenia, bipolar disorder and major depressive disorder

| Saliency map model |  | Schizophrenia (N=231) | Bipolar disorder (N=27) | Major depressive disorder (N=47) |
| --- | --- | --- | --- | --- |
|  |  | chlorpromazine equivalent | Lithium carbonate | Imipramine equivalent |
| Full | *r* | 0.02 | 0.24 | 0.15 |
|  | (*p*) | (1.00) | (1.00) | (1.00) |
| Luminance | *r* | -0.14 | 0.19 | 0.13 |
|  | (*p*) | (0.46) | (1.00) | (1.00) |
| Color | *r* | -0.06 | 0.31 | -0.09 |
|  | (*p*) | (1.00) | (1.00) | (1.00) |
| Orientation | *r* | 0.10 | 0.15 | 0.17 |
|  | (*p*) | (1.00) | (1.00) | (1.00) |

Only Bonferroni-corrected p-values are presented. Raw p-values were multiplied by 12 for correlations between saliency scores and symptom scales.

**References**

1. First M, Spitzer R, Gibbon M, Williams J. Structured Clinical Interview for DSM-IV Axis I Disorders, Clinical Version. American Psychiatric Press: Washington DC, 1997.
2. First M, Spitzer R, Gibbon M, Williams J. Structured Clinical Interview for DSM-IV Axis I disorders, Non-patient Edition. New York State Psychiatric Institute: New York, 1996.
3. Lord, C. et al.Autism Diagnostic Observation Schedule, 2nd edition (ADOS-2), Western Psychological Services, Los Angeles, 2012.
4. Otsubo T, Tanaka K, Koda R, Shinoda J, Sano N, Tanaka S et al. Reliability and validity of Japanese version of the Mini-International Neuropsychiatric Interview. Psychiatry Clin Neurosci 2005; 59: 517-526.
5. Sumiyoshi C, Fujino H, Sumiyoshi T et al. Usefulness of the Wechsler Intelligence Scale short form for assessing functional outcomes in patients with schizophrenia. Psychiatry Res. 2016; 245: 371–378.
6. Fujino H, Sumiyoshi C, Yasuda Y, Yamamori H, Fujimoto M, Fukunaga M, Miura K, Takebayashi Y, Okada N, Isomura S, Kawano N, Toyomaki A, Kuga H, Isobe M, Oya K, Okahisa Y, Takaki M, Hashimoto N, Kato M, Onitsuka T, Ueno T, Ohnuma T, Kasai K, Ozaki N, Sumiyoshi T, Imura O, Hashimoto R; for COCORO. Estimated cognitive decline in patients with schizophrenia: A multicenter study. Psychiatry Clin Neurosci. 2017; 71:294-300.
7. Matsuoka K, Kim Y. Japanese Adult Reading Test. Shinko-Igaku, Tokyo, 2007.
8. Matsumoto J, Fukunaga M, Miura K, Nemoto K, Okada N, Hashimoto N, Morita K, Koshiyama D, Ohi K, Takahashi T, Koeda M, Yamamori H, Fujimoto M, Yasuda Y, Ito S, Yamazaki R, Hasegawa N, Narita H, Yokoyama S, Mishima R, Miyata J, Kobayashi Y, Sasabayashi D, Harada K, Yamamoto M, Hirano Y, Itahashi T, Nakataki M, Hashimoto RI, Tha KK, Koike S, Matsubara T, Okada G, Yoshimura R, Abe O, van Erp TGM, Turner JA, Jahanshad N, Thompson PM, Onitsuka T, Watanabe Y, Matsuo K, Yamasue H, Okamoto Y, Suzuki M, Ozaki N, Kasai K, Hashimoto R. Cerebral cortical structural alteration patterns across four major psychiatric disorders in 5549 individuals. Mol Psychiatry. 2023 28:4915-4923.
